# State of the Art on Toxicological Mechanisms of Metal and Metal Oxide Nanoparticles and Strategies to Reduce Toxicological Risks

**DOI:** 10.3390/toxics9080195

**Published:** 2021-08-23

**Authors:** Victor García-Torra, Amanda Cano, Marta Espina, Miren Ettcheto, Antoni Camins, Emma Barroso, Manel Vazquez-Carrera, Maria Luisa García, Elena Sánchez-López, Eliana B. Souto

**Affiliations:** 1 Department of Pharmacy, Pharmaceutical Technology and Physical Chemistry, Faculty of Pharmacy, University of Barcelona, 08028 Barcelona, Spain; vgarcito8@alumnes.ub.edu (V.G.-T.); acanofernandez@ub.edu (A.C.); m.espina@ub.edu (M.E.); marisagarcia@ub.edu (M.L.G.); 2 Institute of Nanoscience and Nanotechnology (IN2UB), University of Barcelona, 08028 Barcelona, Spain; 3 Networking Research Centre of Neurodegenerative Disease (CIBERNED), Instituto de Salud Carlos III, 28031 Madrid, Spain; mirenettcheto@ub.edu (M.E.); camins@ub.edu (A.C.); 4 Department of Pharmacology, Toxicology and Therapeutic Chemistry, Faculty of Pharmacy, University of Barcelona, 08028 Barcelona, Spain; ebarroso@ub.edu (E.B.); mvazquezcarrera@ub.edu (M.V.-C.); 5 Networking Research Centre of Diabetes and Associated Metabolic Diseases (CIBERDEM), Instituto de Salud Carlos III, 28031 Madrid, Spain; 6 CEB—Centre of Biological Engineering, Campus de Gualtar, University of Minho, 4710-057 Braga, Portugal; 7 Department of Pharmaceutical Technology, Faculty of Pharmacy, University of Coimbra, Pólo das Ciências da Saúde, Azinhaga de Santa Comba, 3000-548 Coimbra, Portugal

**Keywords:** metal nanoparticles, nanoparticles toxicity, reactive oxygen species, nanoparticles functionalization

## Abstract

Metal nanoparticles have been extensively investigated for different types of pharmaceutical applications. However, their use has raised some concerns about their toxicity involving the increase of reactive oxygen species causing cellular apoptosis. Therefore, in this review we summarize the most relevant toxicity mechanisms of gold, silver, copper and copper oxide nanoparticles as well as production methods of metal nanoparticles. Parameters involved in their toxicity such as size, surface charge and concentration are also highlighted. Moreover, a critical revision of the literature about the strategies used to reduce the toxicity of this type of nanoparticles is carried out throughout the review. Additionally, surface modifications using different coating strategies, nanoparticles targeting and morphology modifications are deeply explained.

## 1. Introduction

Nanotechnology, the science of material manipulation at nanoscale level, is believed to be one the most promising fields for biomedical applications. The use of nanomaterials provides unique properties that are not observed at the macroscale level [1].

In this sense, one of the most novel and studied nanostructured systems are metal nanoparticles (MNPs) [2]. In this nanotechnological area, the development of techniques for the controlled synthesis of well-defined metal NPs constitutes an enormous challenge. Metal NPs exhibit unique electronic, magnetic, catalytic and optical properties that are different from those of bulk metals [3]. The exclusive features associated with NPs are responsible for their multifunctional properties and developing interest for their application in various fields such as medical and pharmaceutical industry. In these areas, drug and gene delivery are of high interest. Moreover, certain metals have distinctive properties, such as the antimicrobial properties of gold and silver [4].

Several chemical and physical methods are employed to synthesize these nanoparticles such as chemical reduction, electrochemical synthesis, laser ablation method, mechanical milling, microwave-assisted synthesis or polyol synthesis. Depending on which one is used for the preparation of nanoparticles, there will be differences on their morphology, stability and physicochemical properties [5].

In this sense, one of the problems associated with metal and metal oxide NPs is their possible toxicity [6,7]. Toxicity values are directly related to nanoparticles’ properties such as morphology, size or zeta potential [8]. For instance, nanometric size is considered crucial in nanomaterial toxicity due to their higher surface area leading to a greater reactivity [8]. 

Thus, the selection of preparation methods for metal and metal oxide NPs constitutes a critical parameter to be considered leading to relevant physicochemical properties such as chemical composition, size, solubility, shape or electrical properties, among others, which may be the cause of enhanced toxicity issues to the human body that could result in adverse effects on an organ, tissue or cellular level [9].

The study of the toxicity of metal nanoparticles have received increasing interest and here we describe their toxicity mechanisms, which are of extreme relevance in order to reduce cytotoxic effects in humans.

## 2. Preparation Methods

Two different strategies are used for the preparation of metal and metal oxide NPs, which are bottom up or top down synthesis, depending on the starting material used [10,11]. When the method consists of starting from a bulk material and it is reduced to NPs by different processes, it constitutes a top-down method, whereas if it starts from a single atom or molecules to produce the final formulation it is a bottom-up method [12]. Here, we describe the most commonly used synthesis methods for metal NPs (Table 1).

### 2.1. Laser Ablation

**Figure 1:**
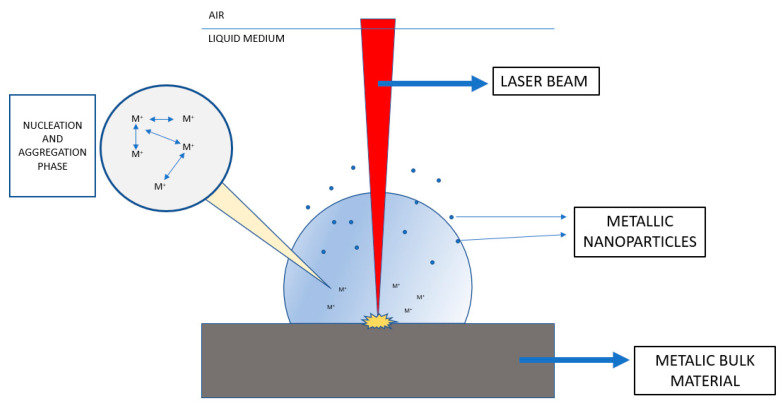
Laser beam synthesis representation (based on [14,15,16]).

This is a top-down process of removing portions from the bulk material by irradiation with a focused laser beam, generating vapor and plasma from target metal immersed in a liquid medium [13]. This vapor-plasma plume with high pressure and temperature is cooled by the surrounding liquid medium, leading to the formation of metal nanoparticles via nucleation and growth steps (Figure 1). The most commonly used vapor lasers are Nd:YAG (Neodymium-doped yttrium aluminum garnet) [14,15,16]. This synthesis method allows a versatile design of the final NPs’ properties by modifying the parameters of the process such as the vapor laser used, time or wavelength of laser pulse, ablation time or liquid medium employed. If these parameters are optimized, this method has the capability of yielding to well-dispersed and stable NPs. This is a simple technique involving an easy experimental procedure with endless possibilities of conditions, which allows fabrication of diverse NPs with desired functions. This technique has been used by several researchers for the preparation of AuNPs, AgNPs or ZnONPs [17,18,19].

### 2.2. Spark Discharge

This is a simple process method and one of the most versatile techniques in the gas phase [20,21]. Nanoparticles are obtained by using electrical discharges between two electrodes separated with dielectric gas. Plasma channel between electrodes is formed with dielectric medium breakdown. The generation of current flow is associated with high temperatures that play an important role in removing material from the electrodes. This is followed by a fast-cooling phase by adiabatic expansion and the nanoparticles are formed by nucleation and growth steps, similar to the laser ablation technique. Interestingly, this method does not require the use of chemical precursors and is considered eco-friendly. It has been used to successfully synthesize AuNPs and AgNPs with a controlled size [22,23]. It was also used to synthesize a mixed metallic nanoparticles (Ag-Cu), (Pt-Au) that are immiscible using a combination of the metals in the electrodes [24].

### 2.3. Evaporation/Condensation

Evaporation/condensation constitutes a synthesis method in gas phase which does not require the use of liquid solvents. In this method the particles are generated by evaporating the material at high temperatures and then decreasing the temperature to condense the metal vapor, forming nanoparticles. This method can be used to control the size of nanoparticles in an accurate manner. Some authors used this method to produce AgNPs with three specific diameters (namely, 50, 90 and 130 nm). The temperature used was 1300–1400 °C. Although AgNPs were successfully obtained, in the collection phase it was observed that some nanoparticles were attached to each other, forming aggregates [25].

Furthermore, other authors were able to eliminate these unwanted aggregates using a silica coating that can avoid flocculation and agglomeration [26]. 

### 2.4. Mechanical Milling

Mechanical milling constitutes a top-down process where the starting material is reduced to a nanostructure by mechanical mixing [27,28,29]. When the mill chamber, which is commonly a hollow cylindrical shell partially filled with grinding bodies made of stainless steel or ceramic materials, starts to rotate about a horizontal axis, it produces a rotational motion of the material placed inside this container causing ball drops from the top of the chamber to the bottom, generating impacts and collisions and finally leading to a homogeneous dispersion of NPs from the target material. The main parameters to be controlled which determine the properties of the final NPs are the raw material used, the size of grinding balls, the degree of filling, the rotation speed and the temperature of the process [27].

This synthesis method allows working at low temperatures, this having the advantage of avoiding degradation of thermolabile materials. Moreover, it does not use organic solvents and it is also convenient in order to be employed for large scale production [30]. The main disadvantages to be overcome are the undesired pollution which comes from the milling media and the difficulty of preparing NPs of a small size range [27].

### 2.5. Electrochemical Synthesis

In the electrochemical synthesis method, it is necessary to use a bulk solution containing metal salts. This technique consists of the electrodeposition of the metal NPs in the substrate surface [31,32]. The main parameters to control in the process are the electrode potential and current density, which will affect the deposition kinetics as well as the nucleation and crystal growth. These parameters are crucial to controlling the properties and morphology of metal NPs. Moreover, nucleation and growth process are important to control in order to synthetize metal NPs with a suitable dispersion [33,34].

### 2.6. Chemical Reduction Method

Chemical reduction constitutes one of the most popular and used synthesis methods, since it is a simple and low-cost method to obtain controlled particle size with low size dispersion. In this process, a bulk solution containing the metal precursor is used and a surfactant and reducing agent are added during the chemical reaction [35]. This process consists of the reduction of the metal cations contained within the metal salts to form metal atoms [36]. The collisions and aggregations between the formed metal atoms and cations result in the formation of clusters. The nucleation is continuously growing to a critical size when the NPs are stabilized. The rate of nucleation growth is crucial to control the shape and size of metal NPs. This rate can be modified using different surfactants, reducing agents or metal precursors, as well as the pH and the temperature at which the reaction is carried out. The metal precursors are usually salts containing the target metal atom. For instance, AgNPs are usually produced using AgNO_3_ as a precursor. A surfactant is used in this process to prevent NP aggregation and to disperse them within the solution. Additionally, NaBH_4_ is widely used as a reducing agent. The use of amine-boranes as reducing agents is being increased [37,38], both for their capability to be used also as stabilizing agents and because they do not require solvent addition [39,40].

One other kind of chemical reduction process is employing polyols that have emerged as a very useful reducing agent. In this process, polyols also act as a solvent of the metal salt precursor. Polyols have some advantages such as their capacity to coordinate particle surfaces, which prevents or minimizes coalescence, its high boiling point and its high viscosity due to the presence of several hydroxyl groups, which allows working at high temperatures and favors the control of particle growth [41]. Several metal NPs have been successfully obtained in the last few years using the polyol synthesis method [42,43,44,45].

### 2.7. Microwave-Assisted Synthesis

In the last several years, the microwave-assisted synthesis method has received increasing interest due to its effective use of dielectric heating that has demonstrated to reduce the time of reaction and the side reactions, which can lead to enhanced chemical yields and reproducibility of the process. Compared to conventional heat conduction methods, the microwave is more efficient in terms of temperature homogeneity and energy used [46]. This technique is usually combined with the previously mentioned methods. For instance, polyol synthesis can be improved with microwave heating [47,48].

### 2.8. Green-Synthesis

Nowadays, the development of metal NPs using green-synthesis methods is being intensely studied [49,50,51,52,53]. Application of green chemistry can reduce the use of hazardous solvents or other toxic compounds, improve the energetic efficiency of reactions and processes, and minimize safety issues. The main strategies are based on plant mediated synthesis where a metal salt precursor is obtained from plants. Plant extracts are also rich on alkaloids, flavonoids, terpenoids or phenolic acids that can reduce metallic ions to metal atoms leading to NP formation [54,55,56]. One of the main advantages of this method is the reduction of human and environmental toxicity, since they are more biocompatible and less harmful for health, and the production methods are eco-friendly, using organic natural sources and reducing energy consumption [49].

**Table 1:** Production methods of metal nanoparticles [12,57].

Synthesis Method	Advantages	Disadvantages
Laser ablation	Simple and effective Easy to modify nanoparticles properties by changing synthesis parameters	The laser path can be blocked by the portions of material released from the surface, causing reduction in ablation rate
Spark discharge	Cost-efficient Environmentally-friendly No impurities	Pure gas is required
Evaporation/condensation	Control of size No solvents used	High energy required
Mechanical milling	Work at low temperatures No solvent used	High energy required Time consuming method Contamination from milling media
Electrochemical synthesis	Simple, fast and inexpensive method Control of size and morphology of nanoparticles	Impurities from liquid media
Chemical reduction method	Simple and effective	Impurities from reaction Toxicity issues of reactive agents
Microwave-assisted synthesis	More efficient use of energy Higher production rates	Less homogeneity of nanoparticles size and morphology
Green synthesis	Eco-friendly Less toxicity Reduction of energy consumption	Use of natural sources Less effective than other methods

## 3. Toxicity Mechanisms of Metal Nanoparticles

A large number of drug delivery formulations based on metal NPs are successfully applied in biomedicine, clinics, cosmetics and pharmaceutical industry. While the inclusion of nanomaterials in several products can enhance their performance, on the other hand, there is growing evidence that the small particle size may also induce undesired side effects. The reduced size at the nanoscale level creates complex physicochemical interactions when exposed to a physiological environment [58].

Consequently, despite some unique advantages associated specifically to metal NPs, potential toxic effects should be considered, both for human administration and for the environment after their application [36]. 

**Figure 2:**
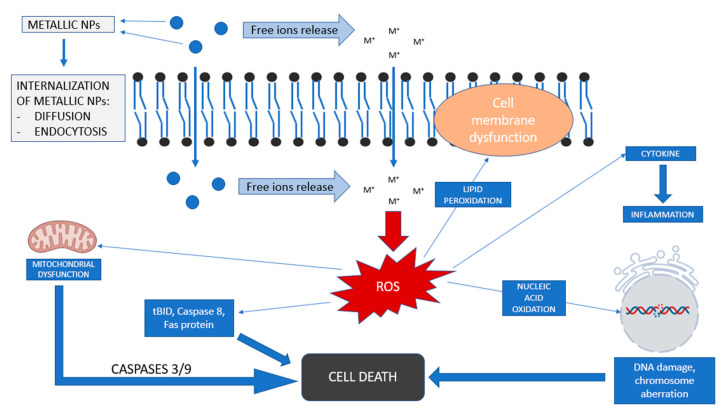
General view of toxicity mechanisms of metallic nanoparticles (based on [59,60]).

The key to understand the toxicity of metal NPs is that their small size allows them to show an increased penetration rate but also to cause alterations in the cellular redox balance leading to increased production of reactive oxygen species (ROS) and thus causing abnormal cellular functionality, leading to cytotoxic effects (Figure 2) [59,60]. Uncontrolled generation of ROS causes harmful effects on cellular structures such as proteins, lipids and nucleic acids, and some evidence shows that this can also be responsible for the progression of several diseases [61,62,63]. The interaction between lipids and reactive species can cause lipid peroxidation. This process is a chain reaction created by free radicals to form lipid hydroperoxides. The accumulation of hydroperoxides and their subsequent decomposition to alkoxyl and peroxyl radicals accelerates the peroxidation of polyunsaturated fatty acids leading to oxidative damage to cell membranes [64]. It has been shown that the consequences of lipid peroxidation include the decrease of lipid fluidity and a subsequent alteration of its permeability and integrity, leading to a functional loss of cell membranes [65].

It has also been shown that ROS can also induce DNA damage by several oxidative reactions with DNA bases leading to mutations. Oxidative DNA damage has been demonstrated to play an important role in the initiation and development of cancer [66,67].

Mitochondria also play and important role in cell damage. The overproduction of ROS cause mitochondrial release of apoptogenic signaling molecules inducing caspase cascade responsible for cell death [68,69]. An additional mitochondrial-independent pathway is also induced by ROS by other signaling molecules such as Caspase 8 or Fas protein.

Thus, to ensure the safe development of NP-containing products, scientific knowledge on potential hazards posed by these nanostructured systems needs to be deepened hand-in-hand with the progress in nanotechnological industry. Reaching this ultimate goal will enable us to avoid, at least, synthesizing potential toxic engineered nanomaterials for commercial use. Moreover, understanding the interaction mechanisms of NPs with cells and their consequences is the first defense in hazard prevention also regarding to accidentally produced NPs due to human activities or derived from natural causes. 

### 3.1. Silver Nanoparticles

Silver NPs (AgNPs) are being increasingly used in many different products. They are well known for their antibacterial activity. However, the potential toxicity of AgNPs constitutes a great concern [70,71,72].

AgNPs can induce toxicity mainly by two different pathways. The first one is the release of a Ag^+^ ions, which are toxic in high concentrations [73,74]. The second one is derived from the nanometric size of AgNPs. These are able to interact with proteins, nucleic acids and carbohydrates of the biological system, modifying their surface properties. However, these surface changes play an important role in the interaction of NPs with cell surface receptors, facilitating the entrance of the NPs via endocytosis. AgNPs’ uptake can also be influenced by their size, shape and concentration [75]. In this area, AgNPs’ concentration-dependent toxicity has been studied in a zebrafish model [76]. In this study, different concentrations (5, 10, 25, 50 and 100 µg/L) of AgNPs were added to zebrafish embryos and incubated for 72 h at 28.5 °C. The mortality of zebrafish embryos increased proportionally to AgNPs’ concentration. The calculated LC_50_ was stablished between 25–50 µg/L and at 100 µg/L only 15–30% were alive. Hearth rate and hatching rate were also induced by AgNP in a dose-dependent manner. In a preclinical study the skin toxicity of AgNPs was evaluated [77]. Three different concentrations of AgNPs (0.34, 3.4 and 34 µg/mL) were topically administered to a pig animal model. Significant differences of focal inflammations and edemas between 0.34 and 34 µg/mL were observed, being the latter the concentration with higher toxic effects [77]. AgNPs can permeate cell membranes and produce higher levels of intracellular Ag^+^, causing cytotoxic and genotoxic effects. The cytotoxic effects of AgNPs have mostly been characterized in terms of oxidative stress, DNA damage and modulation of cytokine production. The cellular uptake of AgNPs can stimulate the production of radical oxygen species (ROS), resulting in oxidative stress. ROS can induce cell death either by apoptosis or necrosis [78,79,80].

Regarding genotoxicity, the increased generation of ROS produced by AgNPs can cause DNA damage by decreasing ATP production, which is associated with mitochondrial damage, impairing energy-dependent DNA repair mechanisms [81]. Direct DNA damage by Ag^+^ or by Ag NPs themselves have also been reported [82,83].

Moreover, in vitro studies have shown the toxic effects of AgNPs in different cells lines. AgNPs have been exposed to murine macrophage cell RAW 264.7 [84], where an increase of cytotoxicity caused by citrate coated 20 nm AgNPs compared with those of 110 nm was observed by causing a more intense acute pulmonary inflammation. The results also showed a reduction of toxicity when AgNPs were coated with PVP avoiding Ag^+^ complexation.

Other studies have also demonstrated size-dependent toxicity of AgNPs. In this sense, Gliga and colleagues used bronchial epithelial cells (BEAS-2B) that were exposed to AgNPs of different average sizes [85]. The results showed a higher cytotoxicity in the smallest size of AgNPs (10 nm) with higher release of free silver ions [86]. Other studies using alveolar epithelial cells (A549) and human epidermal keratocytes (HEKs) revealed a clear influence of the surface properties of AgNPs, the AgNPs coated with carbon being less cytotoxic [87]. Finally, in an interesting study, human mesenchymal cells exposed to AgNPs show a release of interleukins 6 and 8 and vascular endothelial growth factor (VEGF) secretion. Moreover, DNA damage was evaluated with a COMET assay and chromosomal aberration test, showing significant damage after 1 h and a concentration-dependent toxicity [88]. These results showed that the toxicity of AgNPs strongly depends on size and shape, surface properties and concentration.

### 3.2. Gold Nanoparticles

Interest in gold nanoparticles (AuNPs) has increased due to their ease of synthesis and their unique properties with a potential use in drug delivery [89,90] and in biological imaging, which makes them attractive tools for cancer detection and therapy [91,92].

It has been found that the toxicity of AuNPs is closely related to their physicochemical parameters that can influence their biological activity and cellular interactions.

Depending on the synthesis method or functionalization processes, an AuNP’s surface can also be modified, being a crucial factor for toxicity due to the fact that different molecular interaction can occur. For instance, Goodman and co-workers found that modifying the surface charge of AuNPs caused different levels of toxicity. They determined that cationic AuNPs were moderately toxic, whereas anionic AuNPs showed no evidence of toxicity [93]. In contrast, in the study developed by Schaeublin and colleagues [94], both anionic and cationic AuNPs caused toxicity, observing in the anionic AuNPs even higher toxicity. This can be explained by the different synthesis methods, using different chemical groups to modulate the surface charge. Moreover, other studies can be found demonstrating the influence of the ligand agent used to modify AuNPs surface [95].

Currently there are very few published studies that examine the risks and side effects of AuNPs, but they indicate that the cause of toxicity can be related to ROS production. In this sense, Jia et al. found out that when the concentration of AuNPs is increased, the NO-released levels were also elevated thus highlighting that a dose-dependent response in reactive nitrogen species (RNS) exists between RNS and AuNPs [96]. This can be due to the fact that NO can react with superoxide producing peroxynitrite species which can interact with DNA, proteins or lipids via oxidative reactions. This reaction may cause oxidative injury, leading to necroptosis or apoptosis [97]. Li et al. also demonstrated that there is a clear evidence that the presence of AuNPs induce oxidative DNA damage [98]. Moreover, other authors also evidence the direct relationship between AuNPs and ROS concluding that at high doses such as 40–50 μg of AuNPs, a significant ROS induction was detected. Moreover, the latter study also showed that ROS generation can be related to production of TNF-α that could be involved in cell death [99]. Further studies showed that the LC_50_, calculated with Daphnia Magna model, ranged from 65 mg/L to 75 mg/L showing a significance reduction with bimetallic Ag-Au NPs (15 mg/L) [100]. It has also been found that AuNPs can induce autophagy process. Autophagy is a lysosome-dependent degradative pathway that plays an major role in maintaining cellular homeostasis. Ma et al. demonstrated that AuNPs can cause autophagosome accumulation by blocking autophagic flux in a size-dependent manner, showing that 50 nm AuNPs increase the autophagosome accumulation compared to 25 nm and 10 nm [101]. In addition, surface functionalization of AuNPs constitutes a critical aspect to induce autophagy. This has been demonstrated with CTAB-coated Au-nanorods, which possessed higher toxicity than Au-nanorods coated with PSS and PDDAC [102]. This could be interesting for future biomedical applications since recently some researchers have focused on autophagy mechanism as a possible treatment for cancer [103,104].

### 3.3. Copper/Copper Oxide Nanoparticles

As previously mentioned for Au and AgNPs, ROS and RNS production also play an important role in copper/copper oxide nanoparticles (CuNPs/CuONPs) toxicity [105,106,107]. In this area, Sarkar et al. observed that CuNPs exposure induced the production of ROS and NO, which decreases the activity of antioxidant enzymes [108]. A TUNEL assay was performed to confirm that higher levels of ROS also induce a cascade pathway leading to apoptotic cell death in kidney tissue. This study also demonstrated that the presence of CuNPs induces the release of cytochrome C in the cytosol due to the reduction in mitochondrial membrane potential caused by the alteration of the Bcl/Bax ratio. The Bcl family controls the intrinsic apoptotic pathway. The Bcl2 protein has an antiapoptotic effect, contrary to Bax protein which has pro-apoptotic activity, so the Bcl2/Bax ratio is a key factor to control the caspase cascade leading to a programed cell death [109]. The loss of mitochondrial membrane potential induces the loss of level expressions in Caspases 9 and 3 causing cell death via mitochondria-dependent pathway. In addition, the extrinsic pathway where the exposure of CuNPs produced an increase in the cellular levels of Fas protein, caspase 8 and tBID was studied. It suggests the involvement of a mitochondrial-independent pathway. As such, this study suggests the involvement of two apoptotic pathways to control cell death [108]. Those results are in agreement with other studies investigating the induction of oxidative stress in nanotoxicity. Specifically, Assadian et al. found that the generation of ROS results in the alteration levels of glutathione and peroxidase damage to membrane lipids which can cause cell death in human lymphocytes [110]. Moreover, it has also been described that CuNPs induce apoptosis in HepG2 cells via mitochondrial pathway, evidenced by the modification of Bcl2/Bax ratio originating the caspases 9 and 3 activation [111]. Moreover, it has been reported that the LD_50_ of CuNPs (23.5 nm) is around 413 mg/Kg. However, this is still controversial. In this area, an interesting study exposed male rats to several CuNPs concentrations (5, 10 and 100 mg/Kg) and after 2, 7 and 14 days the histological changes and hepatic enzymes level were evaluated. The results show that all concentrations induced toxicity as well as modifications in histopathology of liver and lung tissues [112]. In a different study, CuNPs concentrations ranged between 50 and 200 mg/Kg/day were also applied to rats for 5 days. In this case, the results showed lower hepatotoxicity with 50 and 100 mg/Kg/day than at doses of 200 mg/Kg/day [113].

### 3.4. Zinc/Zinc Oxide Nanoparticles

Zinc and zinc oxide nanoparticles (ZnNPs, ZnONPs) can enter the cell by two different pathways: diffusion of free Zn^2+^ ions dissolved in extracellular medium and direct internalization of ZnNPs [114]. The dissolution of ZnNPs to release Zn^2+^ ions has a strong dependence on pH of the medium. The presence of other components in the medium, the UV radiation, the concentration and NPs size are other factors that can have an influence in the release of Zn^2+^ [115].

ZnO + 2NaOH → Na_2_ZnO_2_ + H_2_O

ZnO + 2HCl → ZnCl_2_ + H_2_O

ZnCl_2_ → Zn^2+^ + 2Cl^−^

The internalization of ZnNPs is carried out via endocytosis mediated by membrane receptor or particular endocytic pathways such as the Claritin-dependent pathway, caveolae-independent pathway or caveoloae-dependent pathway [116].

Free Zn^2+^ generated from ZnNPs has an important effect on toxicity. Studies investigating ZnNPs’ toxicity indicate that a correlation between the concentration of free ions and cell viability exists. Moreover, in this case the consequence is that LDH is released, causing cell membrane damage [117].

Furthermore, the internalized ZnNPs induce the generation of ROS [118], leading to cytokines prompting inflammation that can result in cell death [119].

The toxicity effects of 20 nm ZnONPs were investigated at different doses (5, 50, 100, 300, 1000 and 2000 mg/Kg) administered to rats by oral route [120]. Surprisingly, lower doses showed higher toxicity effects. This might be caused by less agglomeration of ZnO NPs at lower doses that allows them to penetrate into the cells easily [120]. However, future research needs to be focused on lower doses in order to determine the LD_50_ of Zn and ZnONPs.

### 3.5. Iron Oxide Nanoparticles

The toxicity of iron oxide nanoparticles (FeONPs) is closely related to their size, characteristics of the surface, shape and concentration. Fe_2_O_3_ (maghemite) and Fe_3_O_4_ (magnetite) are the most widely used iron materials for NPs synthesis. However, maghemite is preferred to be used since Fe^3+^ can already be found in the human body and is less likely to cause toxic effects. They differ in the oxidation state which modifies their physicochemical properties [121]. The release of iron in cellular medium can produce free radicals by Fenton reaction, in which the combination of iron ions and hydrogen peroxide (H_2_O_2_) generates hydroxyl radicals [122]. The accumulation of hydroxyl radicals can induce lipid peroxidation processes which leads to ferroptosis causing cell death. Recently, iron-based NPs have been studied in cancer therapy due to this phenomenon [123].

Moreover, FeONPs are used as MRI contrast agents at a concentration of 0.56 mg/Kg. Furthermore, concentration-dependent toxicity of FeONPs has been also studied in vitro using murine macrophage cell line J774 at concentrations ranging between 25 and 500 µg/L [124]. Cell viability at 25 µg/L was 95–100% and decreased up to 55–65% at 500 µg/L. Apoptosis index at 25 µg/L was 1.9 and increased up to 26.8 at 500 µg/L [124].

Another in vitro cellular study showed that FeONPs were able to cause cellular membrane damage in erythrocytes, altering their mechanical properties and inducing oxidative stress [125]. The main sources of ROS production are direct generation from their surface, via FeONPs disintegration, from organelle dysfunction or some induction of cell signaling pathways [126,127,128].

Another study showed that internalized FeONPs can induce ROS production in a dose and time-dependent manner. As concentration of FeONPs increased, higher ROS formation was also observed. No further increase in ROS generation was observed at higher concentrations (2 mg/mL, 3 mg/mL), which may be due to saturation in the cellular surface, blocking the uptake of additional FeONPs. Therefore, cellular uptake was at maximum during the first 3 h of exposure, and no increase was found after 4 h, probably due to saturation of the cellular entrance mechanisms [129].

## 4. Strategies to Reduce Metal Nanoparticles Toxicity

In spite of their great potential for pharmacological use, metal NPs are associated with toxicity issues. The main toxicity problems are closely linked with the release of free ions, their reactivity with biological molecules, tendency to agglomerate, or oxidative damage.

Toxicity can be modified depending on the characteristics of metal NPs, such as size and shape, surface charge or composition, which, at the same time, are closely related to the synthesis method. The development and optimization of synthetic routes are crucial to reduce or eliminate the toxicity issue. Moreover, reduction toxicity strategies are summarized in Table 2.

### 4.1. Surface Functionalization

In order to stabilize metal NPs, in addition to enhancing their uptake and biocompatibility, organic functional groups have been used to modify NPs’ surfaces.

**Figure 3:**
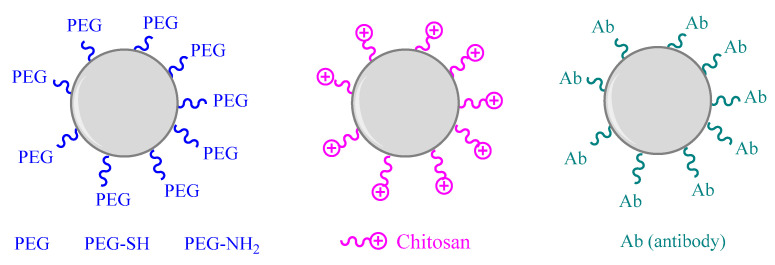
Surface and coating modification strategies of metal and metal oxide nanoparticles (based on [134,138,146]).

One of the most commonly used organic groups for functionalization is polyethylene glycol (PEG), which produces a steric barrier preventing the attachment of phagocytes and shielding the surface from aggregation and opsonization, leading to a prolonged time on the systemic circulation (Figure 3) [8,130,131]. These neutral, flexible, and hydrophilic PEG chains help in minimizing adverse immunological effects [132]. PEG weights range from hundreds to several thousands of Daltons. Additionally, low-weight PEGs are highly soluble in water but PEG solubility decreases with increasing molecular weight [133]. In general terms, to achieve the necessary stealth properties, the most suitable molecular weight of PEG has been reported between 1500–5000 Da [133]. However, with ZnO, TiO_2_ and Cu_2_O it was observed that low-molecular-weight PEG has a smaller steric effect than long-chain PEGs [133].

Moreover, the terminal OH group of PEG may be selectively oxidized to functionalize PEG with various terminal end groups or to attach large ligands [133]. The use of PEG with additional amino (PEG-NH_2_) and thiol (PEG-SH) groups has been advocated as useful strategy [134]. AuNPs with average size ranged between of 1.5 and 5.9 nm have been produced and functionalized with PEG-SH and also with PEG-NH_2_ groups. These NPs show better physical and biological properties for drug delivery applications and no cytotoxic effects were observed in AuNPs-PEG-SH, in contrast with AuNPs-PEG/NH_2_ and AuNPs-PEG [134]. Using AuNPs, it has also been reported that the spacer length of the PEG (the link between the NP surface and the PEG) also plays a major role, especially when fluorescent tags are also attached to the NPs surface [133].

In this area, a significant reduction of cytotoxicity was also observed in ZnNPs functionalized with PEG. The use of PEG reduced the formation of the protein corona, leading to lower toxicity compared to uncoated ZnNPs [135]. Moreover, Wuelfing et al. reported that a monolayer thiol PEGylation of surface metallic AuNPs with octaedral shape by CH_3_O–PEG–SH significantly improved their dispersion stability in aqueous milieu due to the steric repulsion effects of ethered PEG strands [132,136].

In addition, several potential safety concerns have been recently raised from the repeated use of PEG-related products since PEG is not biodegradable. Usually, lower molecular weight PEGs are preferable for biomedical applications since high molecular weight PEG can be acccumulated on some tissues. Moreover, under exogenous stress conditions such as heat, radiation or mechanical forces, PEG showed degradation [132]. Furthermore, PEG’s potential synthetic impurities such as 1,4-dioxane, formaldehyde and cyclic dimer of the ethylene oxide emphasize the usage of a highly technical grade of PEG for biomedical applications [132].

Surface charge is another relevant factor to consider, since it has influence in the NPs’ blood stream solubility and interaction with biological molecules and cell membranes. Therefore, a really interesting computational study showed the effects of AuNPs functionalization with carboxylic groups. These may to be spontaneously protonated on the AuNP’s surface, leading to a more controlled and less disruptive interaction with cell membranes [137]. However, in vivo results will be necessary in order to corroborate this result.

### 4.2. Antibody Functionalization

Antibody-functionalized metal NPs constitute a novel and promising strategy to reduce toxicity by enhancing targeting and effectiveness of drug delivery. In this area, an interesting study using ranibizumab, showed significant improvements in reduction of toxicity in hybrid Au/Fe_3_O_4_NPs. Ranibizumab-coated Au/Fe_3_O_4_NPs were successfully prepared with a non-covalent binding synthesis technique. The MTT assay showed no toxicity of Ranibizumab-coated Au/Fe_3_O_4_NPs [138].

In this area, the use of metal NPs coated with antibodies has a huge potential for cancer treatment, providing better targeting and control of the drug release in specific tumor areas [139].

For instance, AuNPs were conjugated with cetuximab antibody by Li and coworkers to enhance the specificity of AuNPs to human cancer cells through the conjugation to tumor-specific ligands such as EGFR, which is expected to be a promising candidate for cancer therapy [140]. They use the combination of AuNPs charged with H^+^ particles functionalized with antibodies for radiosensitizing therapy. The results showed a good binding capcity between cetuximab and and EGFR in a concentration-dependent manner leading to a rapid and efficient cell uptake. An enhancement of the effect by proton radiation was observed compared with non-targeted AuNPs [141].

### 4.3. Coating Modification

The primary purpose of coating modifications is to reduce metal ion release and agglomerations and avoid oxidation processes.

Within this field, silica-based coatings are the most frequently used. In this sense, ZnNPs have been prepared by Chia et al and coated with silica to prevent the release of Zn^+2^. The results showed that the presence of the silica coating effectively reduced cytotoxicity in addition to retaining the antimicrobial properties of ZnNPs [142]. Another study carried out in vitro using FeONPs coated with thin silica shell in A549 and HeLa cells showed a significant reduction of ROS production in Fe_3_O_4_/SiO_2_ compared to uncoated NPs, leading to a decrease of cytotoxicity [143]. Moreover, a reduction of DNA damage was also observed [143]. Other authors also found that the use of silica coating in AgNPs effectively reduced their toxicity. SiO_2_ was demonstrated to prevent the direct interaction of cells with AgNP surfaces and the liberation of Ag^2+^ ions [144,145].

Chitosan, a natural alkalyne polysaccharide with good biocompatibility and biodegradability, constitutes a widely used compound for NPs coating. This long chain carbohydrate can avoid surface oxidation and control the disintegration of metal NPs, reducing their potential toxicity. Chitosan coating was shown to effectively reduce toxicity of CuNPs in an in vitro study using human A549 cells. This toxicity reduction appears to be linked to decreased ROS generation, suggesting that chitosan coating enhances the release control of copper ions [146]. In a different study, chitosan-coated AuNPs were synthesized to evaluate the uptake, cytotoxicity and immunological responses. The results showed that chitosan can modullate the interaction between AuNPs and proteins in cellular culture medium, modifying cellular responses. The human monocytic cell line was used to assess the uptake and cytotoxicity of chitosan coated AuNPs. Cellular internalization capacity of chitosan coated AuNPs has been compared with citrated funtionalized AuNPs. The positive charge of chitosan has been shown to associate with negatively charged lipid bilayers leading to a greater and faster internalization compared with anionic coatings. Cationic surface has also shown to enhance the interaction with serum proteins since most of them are anionic improving phyisicochemical properties of AuNPs. However, it has to be taken into account the observed cellular inflammatory responses induced by chitosan due to the enhanced particle interaction [146,147]. Figure 3 summarizes the surface and coating modification strategies of metal and metal oxide nanoparticles.

### 4.4. Morphology

#### 4.4.1. Size

The average size of synthesized metal NPs ranges from 1 to 100 nm. This parameter confers different properties compared to the bulk material form. The higher surface area enhances their reactivity, with higher number of molecules interaction in cellular system. Moreover, the reduction of size also allows for enhancing the cellular uptake of NPs, leading to a higher concentration inside the cell [148]. Thus, size plays an important role in determining the toxicity of nanomaterials. Some studies demonstrate the size-dependent toxicity of metal nanoparticles. For instance, a citotoxicity study was carried out exposing different AgNP sizes (20 nm, 80 nm, 113 nm) to RAW 264.7 and L929 cells. The results showed significant differences in ROS production and extracellular LDH activity, being higher in the case of the smallest AgNPs [149].

However, not all studies show higher uptake and toxicity with the smallest NPs. A study developed with AuNPs was performed, exposing different AuNPs sizes (namely, 14, 30, 50, 74 and 100 nm) to cells. Results showed that the greater uptake was obtained by 50 nm AuNPs with no significant difference in toxicity among the populations assesed [150]. On the other hand, an interesting in vivo study showed highest adverse effects in mice of AuNPs with sizes ranging from 8 to 37 nm, while nanoparticles of 3, 5, 50 and 100 nm did not show any cytotoxic effects [151].

Although the majority of the evidence points out a trend towards increased toxicity related with smaller NP size, further studies will be necessary. However, it is sure that size-dependent toxicity has to be taken into account when developing metal and metal oxide NPs in order to reduce their possible side effects.

#### 4.4.2. Shape

**Figure 4:**
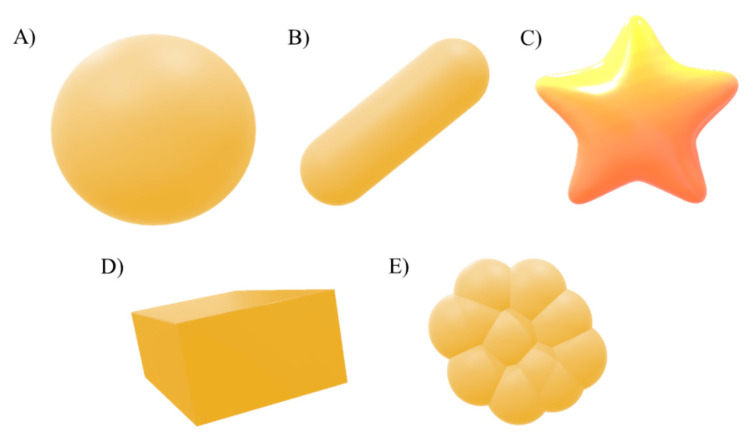
Common shapes of AuNPs; sphere (**A**), rod (**B**), star (**C**) triangles, rods, stars and cubic (**D**) and triangle-like (**E**) prisms (based on [155]).

Shape is also a critical factor contributing to toxic effects. Different NP shapes have influence in the interaction with biological molecules and to cross through biological barriers. Metal NPs can be synthesized in many different shapes such as spheres, triangles, rods, stars and cubic prisms (Figure 4) [149,152].

Nanospheres, nanostars and nanorods based on AuNPs have been synthesized to investigate the influence of shape [153]. Even though the results showed the highest uptake with the nanospherical shape and the lowest with nanostars, the cytotoxicity of nanospheres was also the lowest, suggesting that higher uptake does not always induce higher toxicity [153]. Recently, it has been demonstrated that crystal orientation of metal nanocrystals has a considerable influence in cytotoxicity [154]. The results of this study showed that 100 Pd nanocrystals show less toxicity than 111 Pd nanocrystals. These results seem to be related to the stronger oxygen-binding capacity of 100 Pd nanocrystals compared to 111, resulting in less hydroxyl radical generation and reducing the oxidative damage.

**Table 2:** Summary of reduction of toxicity strategies employed in metal nanoparticles and the techniques used to assed their characterization and therapeutic efficacy (ND: no data available).

Stragey Employed	Type of Metal NP	Functionalitzation Stragey	Physicochemical Characteritzation	In Vitro Studies	In Vivo Studies	References
Surface functionalization	Gold nanoparticles	PEG-SH and PG-NH_2_ groups	TEM/HRTEM, UV-Vis spectroscopy	Citotoxicity assay in SAOS-2 cell line cultivated in McCoy’s 5A medium with 15% heat-inactivated FBS, penicillin and streptomycin	ND	[134]
Surface functionalization	Zinc nanoparticles	PEG	FTIR	Citotoxicity assay in THP-1 immune cells	ND	[135]
Surface functionalization	Gold nanoparticles	Anionic ligands	ND	ND	ND	[137]
Antibody funtionalization	Gold coated magnetite	Antibody raanibizumab	SEM, DLS, XRD, TGA	Citotoxicity test by MTT assay	ND	[138]
Coating modification	Zinc nanoparticles	Silica coating	TEM, XPS, EDX, FTIR	Citotoxicity assessment in both colorectal epithelial cell lines (SW480 and DLD-1)	ND	[142]
Coating modification	Iron oxide nanoparticles	Silica coating	TEM, DLS and potential measurements	Citotoxicity assay in HeLa and A549 cells	ND	[143]
Coating modification	Silver nanoparticles	Silica coating	TEM and SEM images, optical absortion	Toxicity evaluation with *E. coli* bacteria	ND	[26]
Coating modification	Copper nanoparticles	Chitosan coating	XPS, XRD, TEM, DLS	Citotoxicity with human alveolar epithelial cell (A549) using standard MTS assay	In vivo study using mice by nasal administration to investigate inflammatory responses	[146]
Size modification	Silver Nanoparticles	ND	TEM, DLS, Z-potential	Citotoxicity study with murine peritoneal macrophage cell line (RAW 264.7) and L929 fibroblasts	ND	[149]
Size modification	Gold nanoparticles	ND	TEM, ICP-MS	In vitro study with HeLa cells by MTT assay	Mice intraperitoneal injection into BALB/C at a dose of 8 mg/kg/week	[151]
Shape modification	Gold nanoparticles	chitosan	HR Tem images	In vitro study into four cancer cell lines: AGS, HepG2, HT29, HeLa by MTT assay	ND	[156]

## 5. Conclusions

In summary, metal and metal oxide NPs show unique properties that allow them to be a promising tool for the therapeutic treatment of several diseases. Since the use of these nanosystems are of increasing interest, more experts are focusing on the investigation of their abilities to be used for biomedical applications. However, toxicity mechanisms need to be deeply understood in order to be able to reduce their risks. In general terms, the most critical issues are the release of free metal ions, the intracellular uptake and the oxidative reactions leading to inflammatory responses.

The optimization of synthesis methods and the reduction of nanotoxicity are interlocking goals that need to be pursued in parallel, since it is clear that the morphology and physicochemical properties of NPs are closely linked with toxicity. Importantly, metals are not biodegradable materials and it is of crucial relevance to find the most adequate surface functionalization in order to increase their biocompatibility and improve their targeting, trying to avoid the generation of harmful species in body systemic circulation. Optimization of size and shape combined with functionalitzation of NPs by surface coating or ligands such as antibodies attached to their surface consitutes a successful tool to reduce the toxicity of metal NPs.

## Data Availability

Not applicable.
